# “It’s All Under Control” – Description of the Dissociation Between Psychological Symptoms and Autonomic Arousal in People With Obsessive-compulsive Symptoms: A Case-control Study

**DOI:** 10.62641/aep.v53i5.1901

**Published:** 2025-10-05

**Authors:** Sara Guidotti, Alice Fiduccia, Daniele Chirco, Emma Carli, Carlo Pruneti

**Affiliations:** ^1^Department of Medicine and Surgery, Laboratories of Clinical Psychology, Clinical Psychophysiology, and Clinical Neuropsychology, University of Parma, 43121 Parma, Italy

**Keywords:** autonomic arousal, obsession and compulsion, skin conductance, mental health, emotions

## Abstract

**Background:**

Recent literature indicates a significant association between obsessive-compulsive symptoms, depression, anxiety, and suicidal ideation. It appears that psychological symptoms can influence sympathetic activity as well. Our hypothesis suggests that autonomic arousal, as measured by electrodermal activity (EDA), may be lower in people with obsessive-compulsive disorder (OCD) compared to healthy controls.

**Methods:**

To test the experimental hypothesis, eighty-two people diagnosed with OCD were consecutively recruited, and their psychological symptoms were compared to those of a control group along with autonomic arousal. Psychological symptoms were investigated through Symptom Checklist 90-Revised (SCL-90-R). Additionally, baseline, reactivity, and recovery EDA values were recorded during a Psychophysiological Stress Profile (PSP).

**Results:**

The results revealed that people with OCD exhibited significantly higher levels of obsessive-compulsive symptoms (U = 1953.00; *p* < 0.001), depression (U = 2711.00; *p* < 0.001), anxiety (U = 2879.00; *p* < 0.001) as well as suicidal ideation (15.85% in the OCD group and 3.22% in the controls; χ^2^ = 6.03, *p* = 0.01) in comparison with the control group. The global severity index (U = 2317.50; *p* < 0.001) was higher in OCD people as well. However, there were no differences in baseline, reactivity, and recovery EDA levels between the two groups. Correlational analysis indicated that obsessive-compulsive symptoms were negatively associated with reactivity EDA levels (Objective stressor: ρs = –0.29, *p* = 0.03; subjective stressor: ρs = –0.28, *p* = 0.03) in the control group.

**Conclusions:**

These findings highlight a dissociation between subjective and objective measures of mental distress of OCD people. The data suggest that obsessive-compulsive symptoms may play a repressive and suppressive role in managing negative emotions and in the avoidance of autonomic arousal during stress.

## Introduction

Obsessive-Compulsive Disorder (OCD) is a psychological condition characterized by intrusive and distressing thoughts, which individuals struggle to manage. Patients may engage in repetitive rituals or avoid specific triggers to control these thoughts. In agreement with the American Psychiatric Association [1], these efforts are often unsuccessful, leading to further anxiety and fear [2]. The tendency to engage in compulsive behaviors is a key feature of OCD despite experiencing negative consequences. Because of this, OCD is classified among disorders related to self-control and behavioral inhibition [3]. From a neurocognitive perspective, research highlights a reduced ability to inhibit intrusive thoughts and stop repetitive actions. The impairment in inhibitory regulation can contribute to the emotional dysregulation commonly observed in various psychological disorders [4]. People suffering from OCD often experience symptoms of anxiety and depression, as these conditions partially share common brain mechanisms [5].

Additionally, anxiety, depression, and obsessive-compulsive symptoms exhibit high levels of comorbidity, making it very common for individuals with one condition to develop the other two [6, 7, 8]. Thus, it is not surprising that people with OCD often experience symptoms of both depression and anxiety [9, 10]. Patients with OCD often report high levels of depression, anxiety, and psychological stress and frequently use thought suppression as a method to cope with negative emotions [11]. Experiencing intrusive thoughts is a common occurrence even among individuals without clinical disorders. On the other hand, in people with OCD, obsessive thoughts can still feel recurrent, distressing, and uncontrollable [12]. Efforts to suppress cognitive activity often prove ineffective and may inadvertently increase the frequency of intrusive thoughts [13].

Based on these assumptions, researchers generally agree that individuals with OCD exhibit reduced physiological flexibility due to the central nervous system (CNS) consistently functioning in a hyper-aroused state [2]. Consequently, the clinical characteristics of OCD would partially align with those of anxiety disorders, particularly from a neuropsychological and psychophysiological perspective. In both conditions, the challenge lies within the inhibitory mechanisms, which are deemed a risk factor for emotional regulation and the development of psychopathology [14]. More specifically, the inflexible emotional response style that fails to adapt to environmental demands is a common feature of both anxiety disorders and OCD, and it is linked to difficulty in suppressing inappropriate anxious reactions in non-threatening situations. Nevertheless, other studies indicated a different pattern, with some researchers noting that the psychophysiological measures of individuals with OCD did not significantly differ from those of healthy individuals [15].

The tools offered by clinical psychophysiology, including specific biological alterations analysis, can help characterize and distinguish some psychopathological syndromes, which can be useful when clarifying these contradictory results [16]. On top of this, the arousal of the autonomic nervous system (ANS) can be defined by monitoring the trend of the psychophysiological parameter of electrodermal activity (EDA). EDA corresponds to shifts in the skin’s electrical conductance caused by the amount of sweat produced by the eccrine sweat glands present in the hypodermis of hands and feet palms [17]. Emotional and cognitive states were demonstrated to influence EDA as much as the thermoregulatory processes connected to sweating [18]. Thus, EDA is considered a good index of autonomic arousal because it reflects the sympathetic ANS (SANS) activity [19], both at rest and under induced stress. In short, stress reactivity and psychophysical recovery can represent the stress response and the capacity for self-regulation [20]. To summarize, existing research proved that individuals who are depressed and suicidal exhibit electrodermal hypoactivity, while those with anxiety disorders typically manifest autonomic hyperarousal [19, 21].

To our knowledge, the influence of obsessive-compulsive symptoms on the autonomic arousal resulting from the stress response in patients affected by OCD was only partially explored. To illustrate, while some studies report heightened arousal, indicating increased SANS activity, others suggest that the physiological response in individuals with OCD is not significantly different from that of healthy controls [22]. In particular, some research found elevated autonomic arousal in individuals with OCD compared to healthy controls when exploring heart rate [23] and electromyography [24]. Regarding the EDA values, research reported no significant differences in EDA between these two groups [25, 26].

We aimed to provide a significant contribution to the current field of knowledge by testing whether there are significant differences between a group of people with OCD symptoms and a control group, both considering other psychological symptoms (i.e., depression, anxiety, and suicidal ideation) and autonomic arousal (i.e., EDA values). It was hypothesized that a dissociation would be observed between subjective (i.e., psychological symptoms) and objective measures (i.e., autonomic arousal). In particular, the aim was to investigate differences in stress response (i.e., baseline, reactivity, and recovery EDA values) between groups. Therefore, the current study aims to highlight the possible correlation between OCD symptoms on autonomic arousal.

## Materials and Methods

### Procedure

An invitation to participate in the study was displayed on some posters on the bulletin boards of the University of Parma, as well as being distributed through the university mailing list. Participants went to the Clinical Psychology, Clinical Psychophysiology, and Clinical Neuropsychology Laboratories of the Department of Medicine and Surgery to receive a description of the objectives of the study and to provide their consent to participate.

The criteria for participating in the study were: being over 18 years of age; signing the informed consent; no prior history of psychiatric or neurological conditions (i.e., previous head injury, epilepsy, etc.) and/or physical illnesses (i.e., sensory impairments of vision and/or hearing) that could hinder the tests’ administration; and no use of psychotropic medications within the last three months that could affect the ANS (i.e., tricyclic antidepressants; antipsychotics; norepinephrine-dopamine reuptake inhibitors, such as bupropion; serotonin modulators, such as mirtazapine and trazodone; serotonin-norepinephrine reuptake inhibitors, such as venlafaxine and duloxetine.

The study followed a case-control design, as 144 people were consecutively enrolled in the period between January 2023 and December 2024. Eighty-two people with clinically significant OCD symptoms were compared with sixty-two control subjects. The diagnosis of OCD was assessed through the Structured Clinical Interview for DSM-5 Disorders [27].

A licensed clinical psychologist registered on the national list, was available at the end of the experimental procedure for debriefing and to provide individual results through a confidential interview.

The experimental procedures conducted complied with the 1964 Helsinki Declaration of the World Medical Association as well as the 2005 Universal Declaration on Bioethics and Human Rights of UNESCO. This study complies with the Italian privacy law (Legislative decree No. 196/2003).

### Measurements

Psychopathological symptoms were evaluated using the Symptom Checklist-90-Revised (SCL-90-R) [28], a self-report measure that assesses the presence and severity of both internalizing and externalizing psychopathological symptoms experienced by the participant in the week before completing the assessment. It consists of 90 items related to a variety of clinical scales: Somatization (SOM; 12 items), Obsessive-Compulsive (O-C; 10 items), Interpersonal Sensitivity (I-S; 9 items), Depression (DEP; 13 items), Anxiety (ANX; 10 items), Hostility (HOS; 6 items), Phobic Anxiety (PHOB; 7 items), Paranoid Ideation (PAR; 6 items), and Psychoticism (PSY; 10 items). The Global Severity Index (GSI) serves as the most reliable measure of the current level or severity of an individual’s disorder. It combines data about the number of symptoms reported and the intensity of distress experienced. This score is calculated by summing the scores of all 90 items and dividing by 90. Respondents rate the items on a five-point Likert scale representing their distress in the past week: “Not At All” (0), “A Little Bit” (1), “Moderately” (2), “Quite A Lot” (3), and “Extremely Often” (4). The raw score for each scale is derived from the total score divided by the number of items in that scale. The reliability of the symptom dimensions, indicated by Cronbach’s alpha, ranges from 0.67 (PHOB) to 0.87 (DEP), which falls within the acceptable to excellent range. A T-score exceeding 63 on the GSI or at least two symptom dimensions generally indicates a significant clinical psychological issue. Two items of the SCL-90-R probe suicidal ideation. Particularly, items 15, “In the past week, how distressed were you by thoughts of ending your life?” and 59, “In the past week, how distressed were you by thoughts of death or dying?” Suicidal ideation was measured by simultaneously summing the scores of these items. Suicidal ideation was considered present if an affirmative answer was present in at least one item.

A Psychophysiological Stress Profile (PSP) [29] was recorded using the Biograph Procomp Infiniti 5.0 software (Thought Technology Ltd, Montreal, Quebec, Canada) connected to a computer to assess autonomic arousal. The PSP procedure begins by asking the subjects to keep their feet flat on the floor (at 45 degrees) and their arms stretched out along the armrests of the chair in which they are comfortably seated. The room temperature is also controlled (19–21 °C). There are seven recording phases: (1) Baseline, the subject is asked to close their eyes and relax; (2) Stressor 1, the subject is subjected to the Stroop test (computerized version); (3) Rest 1, the subject is asked to relax as much as possible; (4) Stressor 2, the subject is asked to solve a serial subtraction task (for example, consecutively subtract the number 7 starting from 1008); (5) Rest 2, as in Rest 1; (6) Stressor 3, the person is asked to summarize a significant life event; and (7) Rest 3, as in Rest 1. The psychophysiological parameter measured was EDA, which is detected by passing a very low-intensity electric current between two 1 cm^2^ silver circular electrodes applied to the index and middle fingers of the non-dominant hand. The raw value is expressed in micro-siemens (EDAµS) and converted into a percentage value (EDA%). The values ​​of baseline, reactivity, and recovery for both measures (EDAµS and EDA%) were considered. Reactivity and recovery were obtained according to the procedure of Laborde *et al*. [20]. Reactivity is the difference between the stressor phases and the baseline phase (i.e., Stress 1 – Baseline = Reactivity 1; Stress 2 – Baseline = Reactivity 2; and Stress 3 – Baseline = Reactivity 3) while recovery is calculated by subtracting the stressor phase values ​​from the rest phases (i.e., Rest 1 – Stress 1 = Recovery 1; Rest 2 – Stress 2 = Recovery 2; and Rest 3 – Stress 3 = Recovery 3).

### Statistical Analysis

All statistical analyses were conducted using SPSS (Version 28.0.1.0; IBM Corp, Armonk, NY, USA). Descriptive statistics included the computation of the mean (M) and standard deviation (SD) or the median (50th), the 25th, and the 75th quartiles for the variables not respecting the assumptions of normality. Tests for Skewness, Kurtosis, and Kolmogorov-Smirnov were employed to validate the normality of the distribution.

The differences between groups (i.e., OCD group) and controls regarding socio-demographic (i.e., gender, age, marital status, educational level, and occupation), psychological (i.e., obsession-compulsion, depression, anxiety, global severity index, and suicidal ideation), and psychophysiological (i.e., baseline, reactivity, and recovery EDAµS and EDA% values) characteristics were evaluated using Chi-square Test, Independent Samples *T*-Test, or Mann-Whitney U Tests.

A Spearman’s correlation analysis was carried out to investigate the relationship between the psychological and psychophysiological characteristics along with age and gender (coded as 0 = male and 1 = female). The calculation of the ρs (Spearman’s rho) was obtained.

## Results

### Descriptive Analysis of the Sample 

Most of the participants were female (63.89%) while 36.11% were male. The majority of people were single (83.34%) and without children (only 12.5% ​​were parents). Many people were students (72.22%) and had a diploma (50%) or a University degree (47.22%). 22.23% were employed. Demographic characteristics are shown in Table 1.

**Table 1.** **Comparison between OCD and control groups regarding the socio-demographic characteristics**.

Variable		OCD group	Control group	Total sample	*t *or χ^2^	*p*
		(n = 82)	(n = 62)	(n = 144)		
Age,* M (SD)*		27.98 (10.89)	29.10 (9.92)	28.46 (10.46)	0.63	0.26
Sex, *N *(%)					0.83	0.36
	Male	27 (18.75%)	25 (17.36%)	52 (36.11%)		
	Female	55 (38.19%)	37 (25.70%)	92 (63.89%)		
Marital status, *N *(%)					2.72	0.60
	Married/cohabitant	11 (7.64%)	11 (7.64%)	22 (15.28%)		
	Unmarried	70 (48.62%)	50 (34.72%)	120 (83.34%)		
	Separated/divorced	1 (0.69%)	1 (0.69%)	2 (1.38%)		
Children, *N *(%)		11 (7.64%)	7 (4.86%)	18 (12.5%)	2.20	0.53
Education level, *N *(%)					7.28	0.03
	Middle school graduation	2 (1.38%)	2 (1.38%)	4 (2.78%)		
	High school graduation	49 (34.03%)	23 (15.97%)	72 (50%)		
	University degree	31 (21.52%)	37 (25.70%)	68 (47.22%)		
Current occupation, *N *(%)					3.25	0.52
	Student	62 (43.05%)	42 (29.17%)	104 (72.22%)		
	Student + employed	4 (2.78%)	3 (2.08%)	7 (4.86%)		
	Employed	15 (10.42%)	17 (11.81%)	32 (22.23%)		
	Retired	1 (0.69%)	0 (0%)	1 (0.69%)		

OCD, obsessive-compulsive disorder.

### Comparison Between Groups

The Mann-Whitney U Tests conducted on the SCL-90-R T scores revealed that the OCD group reported higher scores on the scales of obsessive-compulsive, depression, anxiety, and general severity index. Furthermore, the frequency of people with suicidal ideation was higher in the group of people with OCD (13 out of 82 people, 15.85%) with respect to controls (2 out of 62 people, 3.22%) (χ^2^ = 6.03, *p* = 0.01).

However, no differences between the EDA values (EDAµS and EDA%) were observed during baseline, reactivity, and recovery phases (Table 2).

**Table 2.** **Comparison between OCD and control groups regarding the psychological and psychophysiological characteristics**.

Variable		OCD group			Control group			U (144)	*p*
		(n = 82)			(n = 62)				
		25th quartile	50th quartile	75th quartile	25th quartile	50th quartile	75th quartile		
SCL-90-R T scores									
	Obsessive-compulsive	68.00	74.66	88.00	45.78	52.44	56.89	1953.00	<0.001
	Depression	58.32	68.77	83.86	47.05	53.18	59.32	2711.00	<0.001
	Anxiety	57.43	66.22	79.73	44.59	50.00	55.41	2879.00	<0.001
	Global Severity Index	62.82	70.00	85.69	46.01	51.48	56.55	2317.50	<0.001
EDAµS									
	Baseline	0.83	1.54	3.10	1.08	1.49	2.83	5870.00	0.76
	Reactivity 1	0.32	0.75	1.50	0.30	0.72	0.29	4371.50	0.62
	Recovery 1	–0.53	–0.17	–0.01	–0.43	–0.17	0.01	5900.50	0.86
	Reactivity 2	0.48	1.05	2.13	0.45	1.04	1.84	4405.50	0.72
	Recovery 2	–0.72	–0.26	–0.01	–0.50	–0.20	–0.06	5935.00	0.97
	Reactivity 3	0.60	1.39	2.48	0.48	1.34	2.15	4284.50	0.40
	Recovery 3	–0.58	–0.28	–0.06	–0.49	–0.15	–0.04	5792.00	0.54
EDA%									
	Baseline	17.37	32.67	62.58	21.49	29.69	56.60	5929.00	0.95
	Reactivity 1	6.32	14.95	30.05	5.94	14.45	25.64	4373.50	0.62
	Recovery 1	–10.80	–3.43	–0.12	–8.60	–3.29	0.17	5900.50	0.86
	Reactivity 2	9.71	20.99	42.77	9.24	20.69	36.72	4419.00	0.76
	Recovery 2	–14.34	–5.16	–0.12	–10.09	–4.12	–1.08	5938.50	0.98
	Reactivity 3	12.10	27.83	49.53	11.59	26.86	44.51	4373.00	0.63
	Recovery 3	–11.63	–5.46	–1.05	–9.64	–2.99	–0.67	5816.50	0.50

SCL-90-R, Symptom Checklist 90-Revised; EDAµS, electrodermal activity in micro-siemens; EDA%, electrodermal activity in percentage value.

### Correlational Analysis

Furthermore, a significant correlation was found examining the relationship between the T score of the obsessive-compulsive scale and reactivity 2 (ρs = –0.29, *p* = 0.03) and reactivity 3 (ρs = –0.28, *p* = 0.03) EDA values in % in the control group. In the OCD group, the same correlation was not confirmed (reactivity 2: ρs = 0.06, *p* = 0.59; reactivity 3: ρs = 0.07, *p* = 0.54).

Lastly, no associations were observed for age and gender.

## Discussion

This study compared the psychological and psychophysiological characteristics of people with OCD to a control group matched for socio-demographic characteristics. The two groups were similar in socio-demographic characteristics, except for the level of education. The reason may be that the sample was mostly composed of students. Specifically, some degree courses in Italy provide for a division of the educational path into a bachelor’s degree and a master’s degree (3 + 2 years) while others are single-cycle (5 or 6 continuous years). The absence of difference between the two groups regarding age suggests that some degree courses may be specifically associated with OCD.

A notable difference concerning psychological comorbidities in OCD people emerged. Specifically, the standardized questionnaire highlighted depressive and anxious levels that were clinically significant (>63 T scores). As a result, the high comorbidity of emotional disturbances with OCD was evident in our sample as well [6, 7, 8], along with suicidal thoughts [30]. These findings are in line with the existing literature that attests that OCD is often associated with a higher psychopathological burden, particularly in terms of comorbidity with depression and anxiety [9, 31]. Conversely, no significant differences were noted when evaluating EDA across the different phases of the psychophysiological stress profile.

Thus, the EDA values of the OCD group were similar to those of the controls [32]. Our results align with earlier research [3, 24], which indicated that the psychophysiological profile of individuals with OCD does not exhibit autonomic hyperarousal. On the contrary, the existence of anxious-depressive symptoms within psychopathological conditions is historically linked with increased hyperarousal [21].

A particularly interesting aspect is the negative correlation between the obsessive-compulsive T score and the EDA% values within the control group, a relationship that was not observed in the OCD group. On the contrary, numerous studies in the literature proved significant differences in autonomic responsiveness among individuals with OCD. Examples include the research by Olbrich *et al*. [33] and Pittig *et al*. [34] who identified a general state of autonomic arousal in patients diagnosed with OCD. The initial study illustrated how a mental task that required engagement of frontal-executive functions (such as inhibitory control) sustained physiological hyperarousal. Pittig *et al*. [34] validated the limited arousal of the parasympathetic branch of the ANS in OCD people, both at rest and during the experimental conditions tested (i.e., relaxation and hyperventilation). As a result, the psychophysiological characteristics of these patients were similar to those with anxiety disorders. Furthermore, the study conducted by Olbrich *et al*. [33] compared an OCD group with a control one and demonstrated that even during a 15-minute resting period, the first group exhibited hyperarousal regarding cardiac function measures (heart rate and heart rate variability).

Other research offered noteworthy contributions by documenting a fragmentation between psychophysiological measures. On top of this, a 2015 study conducted by Whitton and colleagues [24] revealed significant differences between the subjective and objective manifestations of OCD patients. Their findings indicated no association between subjective experience and autonomic arousal (i.e., EDA and surface electromyography recordings) when using body waste images as stimuli in a scientific setting. A similar lack of association between cognitive and autonomic components was observed in a study by Milad and colleagues [35], which compared the responses of OCD patients with those of healthy individuals during a fear extinction task. In line with theories that advocate for the inhibition deficit, the study found that certain brain regions linked to inhibitory control continued to be active even though there was noticeable recovery at the psychophysiological level. Finally, Pöhlchen *et al*. [26] also noted only minor variances in EDA while examining its reactivity in a fear-conditioning experiment.

In line with this viewpoint, the compulsive behaviors displayed by individuals with OCD can be seen as maladaptive coping mechanisms intended to alleviate the distress caused by obsessive thoughts. This finding might explain why numerous studies found little to no significant differences when compared to control groups, indicating a less pronounced emotional response. Nonetheless, it is also feasible that the variations observed by different researchers may be partially attributed to the diverse research methodologies employed and the psychophysiological measures recorded.

To summarize, despite varied findings and differing views presented by researchers, it seems that people with OCD experience autonomic dysfunction. Since the year 2000, Thayer and Lane [36] proposed a significant relationship between cognitive processes and autonomic arousal in individuals with OCD. Specifically, the authors confirmed that a regulatory circuit involving both cortical and subcortical areas plays a role in the management of physiological, emotional, and cognitive functions. Following this model, the prefrontal cortex provides inhibitory control over subcortical pathways, enabling the individual to react to environmental demands in a regulated and adaptive way when necessary.

Despite this, evidence of a mutual relationship between EDA and the visible manifestations of emotion can be traced back to the early days of EDA studies [37]. The effortful control hypothesis proposes that fluctuations in EDA response may illustrate the mental effort required to suppress emotional and aggressive drives. Research examining the performance differences between individuals with labile and stable EDA on tasks that demand cognitive resources offered further support for this hypothesis. If those with labile EDA are preoccupied with managing their emotional and aggressive drives, they would likely perform worse than those with stable EDA on tasks that necessitate extra cognitive effort. It is likely that those with stable EDA, being less mentally burdened, can allocate more cognitive resources when faced with challenges. On the contrary, our findings seem to contradict these assumptions. A possible explanation might be found in understanding the inhibition mechanism associated with emotional regulation. Consequently, individuals with obsessive-compulsive disorders may believe they have control over their emotions and, as a result, feel more assured in directing their energy towards internal cognitive tasks [38].

Our research sheds light on the concealed mechanisms of control and their effectiveness in suppressing emotions triggered by stressors. These results may carry crucial clinical implications. By highlighting the distinction between subjective and objective manifestations, clinicians can gain valuable insights into the best methods for managing patients. Our results indicate that a psychophysiological profile that appears standard, coupled with pronounced psychopathological symptoms, may reveal a robust mechanism for controlling emotional expression.

This finding may indicate that higher levels of obsessive-compulsive symptoms are associated with lower sympathetic activity in response to stress in healthy individuals. This relationship may suggest a more effective autonomic regulation capacity in nonclinical individuals with obsessive-compulsive traits, supporting the “arousal suppression” model as a coping strategy [39]. In contrast, the absence of this correlation in the OCD group may indicate a more pronounced autonomic dysregulation in individuals with clinically relevant OCD symptoms. These findings contribute to a growing body of evidence suggesting that the autonomic response to stress in OCD patients is complex and influenced by multiple factors, including symptom severity, emotion regulation strategies, and comorbidities [40]. This could explain why such a correlation is absent in the OCD group, although there is a negative association between obsessive-compulsive symptoms and EDA values ​​in healthy individuals. A similar pattern had already been described by Huang and colleagues [41], who found a significant correlation between psychophysiological variables of heart rate variability and EDA only in healthy individuals. When individuals with clinically significant symptoms of anxiety and depression were involved, the correlation was not confirmed. In light of these assumptions, this approach could influence therapeutic recommendations aimed at assisting individuals in cognitive restructuring while also improving emotional management and awareness.

Notwithstanding, this study has its limitations. Firstly, the cross-sectional design of the research prevents the establishment of causal relationships between the observed variables. It would be beneficial to replicate the methods and procedures of this study with groups of patients in psychiatric settings in which psychopathological symptoms are more severe. Future studies could also achieve better sample balance, as our group consisted mainly of university students of different types of courses as well as females. Although psychopathological symptoms are more common in women [42], and EDA values are typically lower [43], this correlation was not confirmed in our sample. On the contrary, differences regarding socio-demographic characteristics (i.e., educational level) suggest the need to control for this aspect in future research. Furthermore, another limitation is the absence of standardized tools for measuring autonomic arousal. The demand for standardizing psychophysiological research methods continues to emerge. Improving this aspect of research could reduce the inconsistencies that arise from the varying methodologies employed by different research teams.

This study aimed to investigate the dissociation between subjective and objective dimensions characterizing mental suffering in a group of OCD individuals. A dissociation was observed between mental distress reported in questionnaires and autonomic arousal assessed by psychophysiological measures. In particular, while OCD patients manifested a level of autonomic reactivity comparable to that of control subjects, they reported significantly higher levels of anxiety, depression, and suicidal ideation. Furthermore, the correlation analysis between EDA values ​​and various psychological variables indicated that obsessive-compulsive symptoms play a role in the regulation of emotional responses, as higher levels of obsessive-compulsive traits were associated with lower autonomic arousal in healthy individuals. On the contrary, the absence of such a correlation in the OCD group suggests a more pronounced autonomic dysregulation in these patients, which may be exacerbated by comorbid emotional disorders such as anxiety and depression. These findings contribute to a growing body of evidence suggesting that the autonomic response to stress in people with OCD is influenced by multiple factors, including symptom severity, emotion regulation strategies, and comorbid conditions. Our findings highlight the importance of understanding control mechanisms in individuals with OCD, particularly concerning the complex interplay between subjective experiences and physiological responses. This highlights the need to look at both subjective and objective dimensions of distress when evaluating and treating patients with OCD. Given the potential impact of mental health disorders on physiological health, identifying conditions with poor mind-body integration is essential for effective intervention and management strategies.

Finally, these findings suggest that treatment recommendations for patients with OCD should address not only cognitive restructuring but also emotional regulation and awareness, particularly in the context of comorbid conditions. Future research should aim to refine psychophysiological assessment tools and methods to better understand the autonomic dysregulation observed in OCD, potentially improving both diagnostic and therapeutic approaches.

## Conclusions

Our study aimed to investigate the dissociation between subjective and objective dimensions characterizing mental suffering in a group of OCD individuals. A dissociation was observed between the psychological symptoms assessed through the standardized questionnaire and the autonomic arousal measured through the psychophysiological stress profile. In particular, while OCD patients were characterized by a level of SANS activity comparable to that of control subjects, they reported significantly higher levels of depression, anxiety, and suicidal ideation. Furthermore, the correlation analysis between EDA values ​​and psychological characteristics indicates that obsessive-compulsive symptoms might play a role in the regulation of emotional responses, as higher levels of obsessive-compulsive traits were associated with lower sympathetic activity in healthy individuals. On the contrary, the absence of such a correlation in the OCD group suggests a more pronounced autonomic dysregulation in these patients, which may be exacerbated by comorbid emotional disorders, such as anxiety and depression. These findings contribute to a growing body of evidence suggesting that the autonomic response to stress in patients with OCD is influenced by multiple factors, including symptoms’ severity, emotion regulation strategies, and comorbid conditions. Our findings highlight the importance of understanding control mechanisms in individuals with OCD, particularly concerning the complex interplay between subjective experiences and physiological responses. This highlights the need to take into account both subjective and objective dimensions of distress when assessing and treating patients with OCD.

## Availability of Data and Materials

Data to support the findings of this study are available on reasonable request from the corresponding author.
